# Characterization of the complete mitochondrial genome of *Longicollum pagrosomi* yamaguti, 1935 (Palaeacanthocephala: Echinorhynchida) in cultured large yellow croaker (*Larimichthys crocea*) and its phylogenetic implications

**DOI:** 10.1017/S003118202510036X

**Published:** 2025-08

**Authors:** Zhongjie Ren, Xiaoao Yang, Lihua Jiang, Denghui Zhu, Zhen Tao, Yanjie Wang, Peipei Fu, Rui Song

**Affiliations:** 1 National Engineering Research Center of Marine Facilities Aquaculture, Marine Science and Technology College, Zhejiang Ocean University, Zhoushan, P. R. China; 2 Zhoushan Fishery Breeding and Hatching Innovation Center, Zhoushan, P. R. China; 3 National Engineering Laboratory of Marine Germplasm Resources Exploration and Utilization, Marine Science and Technology College, Zhejiang Ocean University, Zhoushan, P. R. China; 4 School of Fisheries, Zhejiang Ocean University, Zhoushan, P. R. China; 5 Hunan Fisheries Science Institute, Changsha, P. R. China

**Keywords:** *Longicollum*, mitogenome, phylogeny

## Abstract

A species of acanthocephalan collected from the hindgut of *Larimichthys crocea* was identified as *Longicollum pagrosomi* Yamaguti, 1935 based on morphological characteristics. The complete mitochondrial genome of this parasite was sequenced. The mitogenome exhibited a circular structure with a total length of 14 632 bp, containing 12 protein coding genes (PCGs), 2 ribosomal RNAs (rRNAs), 22 transfer RNAs (tRNAs) and 2 major non-coding regions. The most frequently used start codon was GTG, and the most abundant amino acid was valine. The phylogenetic analyses of the mitogenome using Bayesian inference and maximum likelihood methods showed that the genus *Longicollum* formed a sister clade to the genus *Pomphorhynchus*, supporting the monophyly of *Pomphorhynchus*. This study reported a new host for *L. pagrosomi* and revealed the first complete mitogenome sequence of the genus *Longicollum*.

## Graphical abstract

**Figure:**
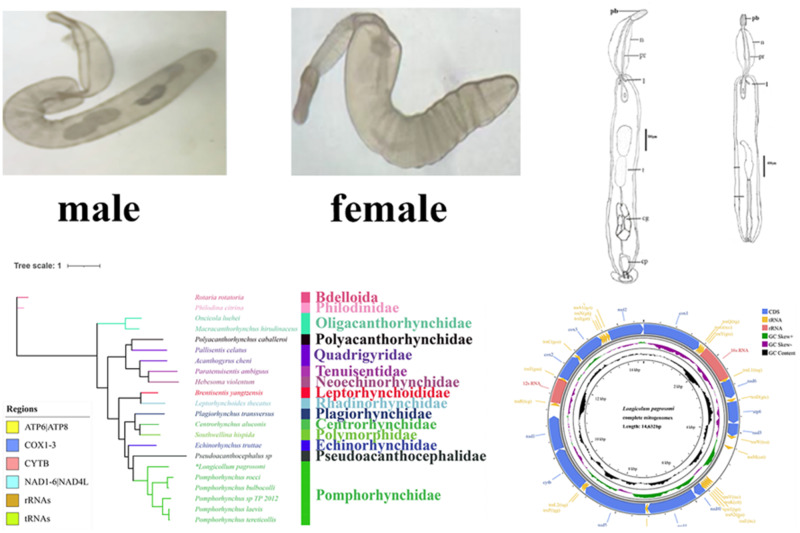

## Introduction

The large yellow croaker (*Larimichthys crocea*) is a high-value species in China’s mariculture industry, with an annual production exceeding 281 000 tons in 2023 (Liu, 2024). However, the sustainable development of *L. crocea* aquaculture is hindered by several diseases (Tang *et al*. 2022), particularly parasitic pathogens, such as *Cryptocaryon irritans* (Zuo et al., 2012), *Neobenedenia melleni* (Yang et al., 2004) and *Trypanosoma larimichthysi* (Yang et al., 2025).

## Material and method

### Sample collection

During a helminthological survey of the large yellow croaker, *Longicollum pagrosomi* (Yamaguti, 1935) was collected from the hindgut of cage-cultured *L. crocea* in the Sanduao Bay in Ningde, Fujian Province. A total of 11 acanthocephalans, including 6 males and 5 females, were fixed in 70% ethanol for morphological identification.

### Morphological identification

The parasite samples were processed following the protocol described by Fu et al. (2019). After hydration, the specimens were stained with ferric hydrochloric acid carmine, differentiated in 70% acid ethanol, dehydrated through a graded ethanol series and clarified in xylene. The samples were mounted with Canada balsam and examined under a light microscope (Olympus, DP72, Japan). Images were captured for further analysis, and morphological measurements (Table S1) were conducted using ImageJ software. All measurements were expressed in millimetres unless otherwise specified.

### DNA extraction, primer designed, PCR amplification, sequencing of mitogenome and sequence annotation

To obtain the mitogenome of *L. pagrosomi*, genomic DNA was extracted from a single specimen using the Tissue Cell Genome Kit. Primers (Table S2) targeting conserved mitochondrial regions were used to amplify short fragments of 16S, *nad*4, *nad*5, *cyt*b, and 12S. Specific primers were then designed to amplify the remaining sequence. PCR conditions followed those described in a previous study (Song et al., 2019). The PCR products were sequenced using a primer-walking strategy at Sangon Biotech. The mitogenomic sequences were manually assembled using DNASTAR v7.1 software (Burland, 2000) and annotated according to the procedures described by Li et al. (2019). Raw sequences were imported into the online software MITOS (http://mitos.bioinf.uni-leipzig.de) to determine approximate gene boundaries. The precise positions of protein-coding genes (PCGs) were identified by searching for open reading frames (ORFs) using genetic code 5 (invertebrate mitochondrion). A majority of transfer RNAs (tRNAs) were identified using MITOS and RNAfold WebServer (http://rna.tbi.univie.ac.at), with the remaining tRNAs identified by alignment with other acanthocephalan species. The boundaries of the 2 ribosomal RNAs (rRNAs), *rrn*L and *rrn*S, were determined by comparing them with homologous sequences. Codon usage and relative synonymous codon usage (RSCU) for the 12 PCGs were calculated using PhyloSuite software (Zhang et al., 2020). AT and GC skew values were calculated using the following formulas: AT-skew = (A – T)/ (A + T) and GC-skew = (G – C)/(G + C). The circular map of the *L. pagrosomi* mitogenome was visualized using the CGView server (http://cgview.ca). The secondary structure of tRNAs and rRNAs were displayed using Adobe Photoshop CC (Figure S1 and S2).

### Phylogenetic analyses

Phylogenetic analyses based on the newly sequenced mitogenome and 23 acanthocephalan mitogenomes available in GenBank (Table S3) were conducted. *Rotaria rotatoria* Pallas, 1776 (NC013568.1) and *Philodina citrina* Lansing, 1947 (FR856884.1) were selected as outgroups. Fasta files for the sequences, including 12 PCGs, 22 tRNAs and 2 rRNAs, were retrieved from GenBank using PhyloSuite, followed by multiple sequence alignment in MAFFT (Katoh et al., 2002) and sequence concatenation. The optimal partitioning schemes and models were determined using PartitionFinder 2 (Lanfear et al., 2017). Bayesian inference (BI) analysis was conducted using MrBayes 3.2.7 (Ronquist et al., 2012) with default settings and 2, 000, 000 metropolis-coupled MCMC generations. Maximum-likelihood (ML) analysis was performed using IQ-TREE (Nguyen et al., 2014) with 50 000 ultrafast bootstraps.

## Result

### Morphological description

The morphology of the acanthocephalan was shown in Figure S3. The presoma and metasoma (trunk) of the parasite were creamy white and taupe, respectively. The trunk was cylindrical, with an elongated neck exhibiting distinct expansion. The proboscis was short, club-shaped and gradually widened toward the anterior region, adorned with 10–14 spiral, longitudinal rows, each containing 11–16 hooks.

### Chracterization of the mitochondrial genome of *L. pagrosomi*

**Figure 1.**
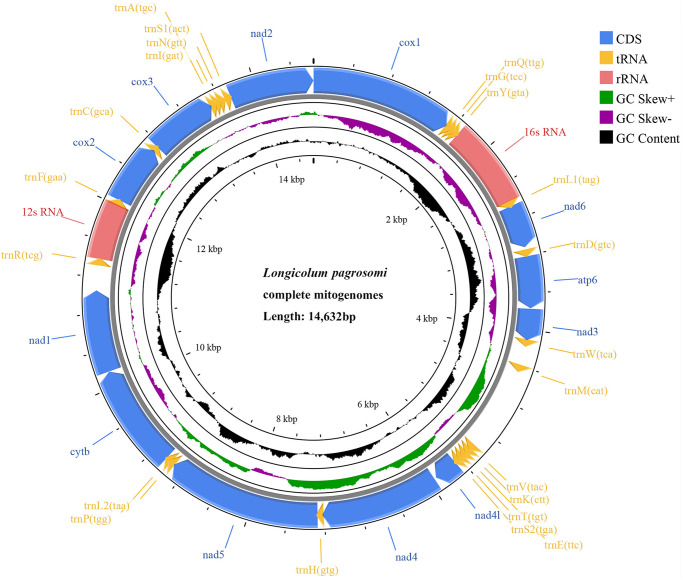
Map of the complete mitogenomes of *longicollum pagrosomi*. 12 protein-coding genes (12) are shown in blue, rRNAs (2) in pink, and tRNAs (22) in yellow.

**Figure 2.**
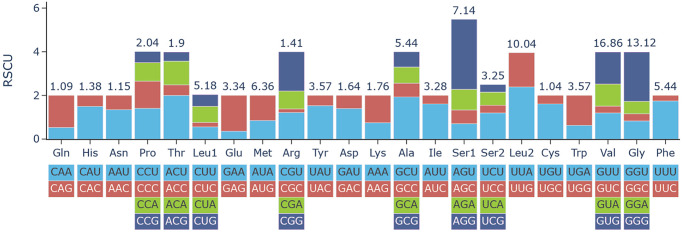
Relative synonymous codon usage (RSCU) of the complete mitogenomes of *L. pagrosomi*. Codon families are labelled on the *x*-axis. Values on the top of the bars refer to amino acid usage.

**Figure 3.**
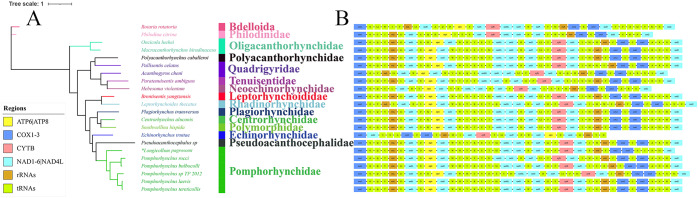
Phylogeny and gene order of the acanthocephalans. (A) Phylogenetic tree was constructed using the Bayesian inference method for almost complete genomic datasets (36 genes: 12 PCGs, 2 rRNAs and 22 tRNAs). (B) Linear comparison of genome order.

The circular duplex mitogenome of *L. pagrosomi* was 14 632 bp in size (GenBank accession number: OR215045) and contained all 36 typical metazoan genes, including 12 PCGs, 22 tRNAs and 2 rRNAs, but lacked the *atp*8 gene (Figure 1). All genes were transcribed from the same strand, and the genome featured 11 overlapping regions (Table 1). The base composition of the mitogenome was as follows: A: 21.63%, T: 34.16%, C: 10.91% and G: 33.30%, indicating an AT bias. The overall nucleotide composition of the complete mitogenome was skewed away from A in favour of T, and strongly biased toward G, with an AT skew of −0.225 and a GC skew of 0.672 (Table 2). The concatenated length of the 12 PCGs was 10 405 bp, with an average A + T content of 54.79%, ranging from 51.34% in *atp*6 to 56.51% in *cyt*b (Table 2). The most frequently used start codon was GTG (observed in eight PCGs), followed by TTG (in two PCGs). The most common stop codon was TAG (found in five PCGs), while TAA was used by *nad*2, *nad*6 and *cox*3 (Table 1). Codon usage and RSCU were presented in Figure 2 and Table 3. Among the PCGs of *L. pagrosomi*, valine (16.86%), leucine (15.22%), glycine (13.12%) and serine (10.39%) were the most abundant amino acids, while cysteine (1.04%), glutamine (1.09%) and asparagine (1.15%) were relatively rare. The higher T content (34.19%) in the 12 PCGs correlated with a higher frequency of T-rich codons, including TTA for leucine (6.07%), GTT for valine (5.06%) and TTT for phenylalanine (4.75%). All 22 tRNAs were identified in the *L. pagrosomi* mitogenome, with a total length of 1255 bp, and sizes ranging from 44 bp (*trn*C) to 72 bp (*trn*L1) (Table 1). The 2 rRNAs, *rrn*L and *rrn*S, were 908 bp and 615 bp in length, respectively, with A + T contents of 61.46% and 63.25%. The *rrn*L (16S) gene was located between *trn*Y and *trn*L1, while *rrn*S (12S) was positioned between *trn*F and *trn*R. This genes arrangement was consistent with other members of the Pomphorhynchidae family (Figure 3).

**Table 1.** The organization of the mitochondrial genome of *L. pagrosomi*

Gene	Direction	Position		Length (bp)	Start/Stop codon	Intergenic nucleotide (bp)	Anticodon
*cox1*	+	1	1533	1533	GTG/TAG	2	
*trnG*	+	1536	1588	53		−6	TCC
*trnQ*	+	1583	1633	51		−4	TTG
*trnY*	+	1630	1683	54		−1	GTA
*16S*	+	1683	2590	908		0	
*trnL1*	+	2591	2662	72		−16	TAG
*nad6*	+	2647	3111	465	GTG/TAA	31	
*trnD*	+	3143	3211	69		−1	GTC
*atp6*	+	3211	3771	561	TTG/TAG	5	
*nad3*	+	3777	4116	340	ATG/GAT	0	
*trnW*	+	4117	4178	62		220	TCA
*trnM*	+	4399	4457	59		937	CAT
*trnV*	+	5395	5452	58		0	TAC
*trnK*	+	5453	5507	55		0	CTT
*trnE*	+	5508	5559	52		0	TTC
*trnT*	+	5560	5614	55		−3	TGT
*trnS2*	+	5612	5664	53		−3	TGA
*nd4L*	+	5662	5923	262	GTG/GTT	22	
*nad4*	+	5946	7223	1278	GTG/TAG	0	
*trnH*	+	7224	7274	51		0	GTG
*nad5*	+	7275	8940	1666	GTG/TTT	0	
*trnL2*	+	8941	9006	66		−12	TAA
*trnP*	+	8995	9046	52		0	TGG
*cytb*	+	9047	10168	1122	GTG/TAG	2	
*nad1*	+	10171	11054	884	TGT/TTT	280	
*trnR*	+	11335	11393	59		0	TCG
*12S*	+	11394	12008	615		0	TAC
*trnF*	+	12009	12059	51		0	GAA
*cox2*	+	12060	12713	654	GTG/TAG	2	
*trnC*	+	12716	12759	44		3	GAT
*cox3*	+	12763	13494	732	TTG/TAA	−2	
*trnA*	+	13493	13545	53		−10	TGC
*trnI*	+	13536	13601	66		−9	GAT
*trnN*	+	13593	13649	57		17	GTT
*trnS1*	+	13667	13722	56		0	ACT
*nad2*	+	13723	14631	909	GTG/TAA	0

**Table 2.** Nucleotide composition and skewness of different elements of the mitogenome of *L. pagrosomi*

Region	Size (BP)	A (%)	T (%)	G (%)	C (%)	A + T (%)	G + C (%)	AT-skew	GC-skew
Mitogenome	14 632	21.63	34.16	33.30	10.91	55.79	44.21	−0.225	0.672
*cox1*	1533	20.87	35.55	29.75	13.83	56.42	43.58	−0.260	0.535
*cox2*	654	20.18	33.34	35.93	10.55	53.52	46.48	−0.246	0.706
*atp6*	561	17.83	33.51	34.22	14.44	51.34	48.66	−0.305	0.578
*cox3*	732	20.22	35.11	35.52	9.15	55.33	44.67	−0.269	0.742
*nad3*	340	18.82	37.35	30.88	12.94	56.17	43.82	−0.330	0.581
*nad1*	883	18.46	36.81	33.52	11.21	55.27	44.73	−0.332	0.666
*nad5*	1666	19.93	33.61	36.61	9.84	53.54	46.45	−0.256	0.731
*nad4*	1278	18.54	35.29	38.34	7.82	53.83	46.16	−0.311	0.796
*nad4l*	262	19.08	36.26	37.40	7.25	55.34	44.65	−0.310	0.806
*nad6*	465	19.78	32.90	34.84	12.47	52.68	47.31	−0.249	0.642
*cytb*	1122	20.59	35.92	31.82	11.68	56.51	43.50	−0.271	0.633
*nad2*	909	21.34	33.55	33.55	11.55	54.89	45.10	−0.222	0.656
*NCR1*	222	31.53	33.78	25.68	9.01	65.31	34.69	−0.034	0.649
*NCR2*	937	13.87	38.42	39.17	8.54	52.29	47.71	−0.469	0.782
*NCR3*	280	10.36	27.50	29.29	32.86	37.86	62.15	−0.453	−0.122
*12S*	615	31.22	32.03	25.85	10.89	63.25	36.74	−0.013	0.579
*16S*	908	30.29	31.17	26.43	12.11	61.46	38.54	−0.014	0.542
tRNAs	1255	27.09	30.84	30.52	11.55	57.93	42.07	−0.065	0.622
rRNAs	1522	30.61	31.54	26.22	11.63	62.15	37.85	−0.015	0.556
PCGs	10 405	20.60	34.19	33.14	12.07	54.79	45.21	−0.248	0.636

**Table 3.** The codon number and relative synonymous codon usage in the mitochondrial genomes of the *L. pagrosomi*

Codon	Count	RSCU	Codon	Count	RSCU
**GCU**	**91**	**1.93**	AAC	13	0.65
GCC	30	0.63	CCU	25	1.41
GCA	35	0.74	CCC	22	1.24
GCG	33	0.7	CCA	15	0.85
UGU	29	1.61	CCG	9	0.51
UGC	7	0.39	CAA	10	0.53
GAU	40	1.4	CAG	28	1.47
GAC	17	0.6	CGU	15	1.22
GAA	21	0.36	CGC	2	0.16
**GAG**	**95**	**1.64**	CGA	10	0.82
**UUU**	**165**	**1.75**	CGG	22	1.8
UUC	24	0.25	UCU	54	1.2
**GGU**	**95**	0.83	UCC	16	0.35
GGC	38	0.33	UCA	27	0.6
GGA	65	0.57	UCG	16	0.35
**GGG**	**258**	**2.26**	AGU	32	0.71
CAU	36	1.5	AGC	28	0.62
CAC	12	0.5	AGA	43	0.95
**AUU**	**92**	**1.61**	**AGG**	**145**	**3.21**
AUC	22	0.39	ACU	33	2
AAA	23	0.75	ACC	8	0.48
AAG	38	1.25	ACA	18	1.09
**UUA**	**211**	**2.39**	ACG	7	0.42
**UUG**	**138**	**1.57**	**GUU**	**176**	**1.2**
CUU	49	0.56	GUC	46	0.31
CUC	18	0.2	**GUA**	**148**	**1.01**
CUA	65	0.74	**GUG**	**216**	**1.47**
CUG	48	0.54	UGA	39	0.63
**AUA**	**94**	**0.85**	**UGG**	**85**	**1.37**
**AUG**	**127**	**1.15**	**UAU**	**95**	**1.53**
AAU	27	1.35	UAC	29	0.47

### Phylogeny and gene order

Phylogenetic analysis of the concatenated 22 mitochondrial genes, using both ML and BI methods, produced identical topologies with strong statistical support for most nodes. Therefore, only the BI tree was shown (Figure 3). The results indicated that the newly sequenced mitogenome of *L. pagrosomi* from large yellow croaker formed a sister clade with *Pomphorhynchus* species, supporting the monophyly of the *Pomphorhynchus* genus. The mitochondrial gene arrangement in acanthocephalan species was generally conserved, with the arrangement of the 12 PCGs and 2 rRNAs being consistent (Figure 3).

## Discussion

In this study, we collected an acanthocephalan species from the large yellow croaker. Morphological analysis clearly identified the specimens as *L. pagrasomi* (Yamaguti, 1935; Wang et al., 1993; Li et al., 2017a; Cheng et al., 2022), based on the number of longitudinal rows of proboscis hooks, the number of hooks per longitudinal row, the shape and length of the proboscis hooks, trunk size, cement glands and proboscis receptacles (Table S1). However, the color of the samples was taupe, differing from previous descriptions (Kim et al., 2011; Cheng et al., 2022). The body colour of this parasite has been reported to vary, including white, orange, red, green and black (Ha et al., 2017; Cheng et al., 2022), and the variation in colour could be attributed to differences in the host type, possibly related to host-derived pigments and dietary composition.

The genus *Longicollum* includes 13 nominal species, with only 2 species, *Longicollum alemniscus* and *L. pagrosomi*, reported from Chinese waters (Wang et al., 1993). *Longicollum pagrosomi* parasitizes the intestine of marine fish, with a broad host range that includes Sparidae (Yamaguti, 1935; Wang et al., 1993), Oplegnathidae (Li et al., 2017a) and Lutjanidae (Cheng et al., 2022). In this study, *L. pagrosomi* was reported from a new host, Sciaenidae, *L. crocea*. The 4 species of Perciformes fish hosting *L. pagrosomi* were all collected from the East China Sea and Japan, indicating that *L. pagrosomi* has a wide spectrum of definitive hosts.

In the order Echinorhynchida, only 14 acanthocephalan species from 8 different families have their mitogenomes sequenced (Steinauer et al., 2005; Weber et al., 2013; Song et al., 2019; Muhammad et al., 2020; Gao et al., 2023; Zhao et al., 2023; Xie et al., 2024). No mitogenomic data for *Longicollum* species had been reported previously. This study presented the first mitogenome of *L. pagrosomi*, exhibiting several common features of Acanthocephala. All genes in the mitogenomic structure were encoded on the same strand, a characteristic typical of Acanthocephala (Song et al., 2019). The newly sequenced mitogenome lacks the *atp*8 gene, a trait common to parasitic flatworms (Le et al., 2002). Additionally, the mitogenome of *L. pagrosomi* exhibited several unique features, including an overall A + T content of 55.79%, the lowest reported among mitogenomes of the Echinorhynchida (Xie et al., 2024). Leucine is typically the most abundant amino acid in the PCGs of fish acanthocephalan mitogenomes (Song et al., 2019; Muhammad et al., 2023; Xie et al., 2024). While, *L. pagrosomi* predominantly uses valine (16.86%). The gene order of 12 PCGs and 2 rRNA in *L. pagrosomi* matches that observed in other fish acanthocephalans, including *cox*1, *rrn*L, *nad*6, *atp*6, *nad*3, *nad4*L, *nad*4, *nad*5, *cty*b, *nad*1, *rrn*S, *cox*2, *cox*3 and *nad*2 (Song et al., 2019; Muhammad et al., 2023; Zhao et al., 2023; Xie et al., 2024). Only a few tRNAs translocations (*trnR, trnM* and *trnI*) were detected in the mitogenome of *L. pagrosomi* (Figure 3), and the arrangement of tRNA gene in this mitogenome differs from all known acanthocephalan mitogenomes.

Phylogenetic analysis based on mitochondrial genes (12 PCGs + 22 tRNA + 2 rRNA) from this study, using both ML and BI methods, supported the monophyly of the genus *Pomphorhynchus*, consistent with the current taxonomy of the genus (Amin 2013). However, the tree topology based on the 18S, ITS and *cox*1 sequences of *Pomphorhynchus zhoushanensis* Li, Chen, Amin & Yang, 2017, using maximum parsimony (MP) and ML methods showed that *Pomphorhynchus* was a paraphyletic group (Li et al., 2017b). The discrepancy between these results indicates that the taxonomic status of *P. zhoushanensis* remains uncertain and requires further investigation.

## Conclusion

In summary, *L. crocea* represents a new host for *L. pagrosomi*, thereby expanding its host range within Perciformes. This study provides the first mitochondrial genome of *Longicollum* and supports the monophyly of *Pomphorhynchus* while raising doubts about the classification of *P. zhoushanensis*. The findings contribute significantly to the genetic data available for acanthocephalans.

## Supporting information

Ren et al. supplementary material 1 Ren et al. supplementary material

Ren et al. supplementary material 2 Ren et al. supplementary material

Ren et al. supplementary material 3 Ren et al. supplementary material

Ren et al. supplementary material 4 Ren et al. supplementary material

Ren et al. supplementary material 5 Ren et al. supplementary material

Ren et al. supplementary material 6 Ren et al. supplementary material

## Data Availability

The newly generated mitochondrial genome of *Longicollum pagrosomi* have been submitted to the NCBI GenBank database with accession numbers OR215045.

## Supplementary material

The supplementary material for this article can be found at https://doi.org/10.1017/S003118202510036X.
