# Supplementary figures and images for: Characterization of the complete mitochondrial genome of Longicollum pagrosomi yamaguti, 1935 (Palaeacanthocephala: Echinorhynchida) in cultured large yellow croaker (Larimichthys crocea) and its phylogenetic implications
Source: Parasitology. 2025 Jul 1;152(9):951–7. doi: 10.1017/S003118202510036X (PMC12644934; doi:10.1017/S003118202510036X)

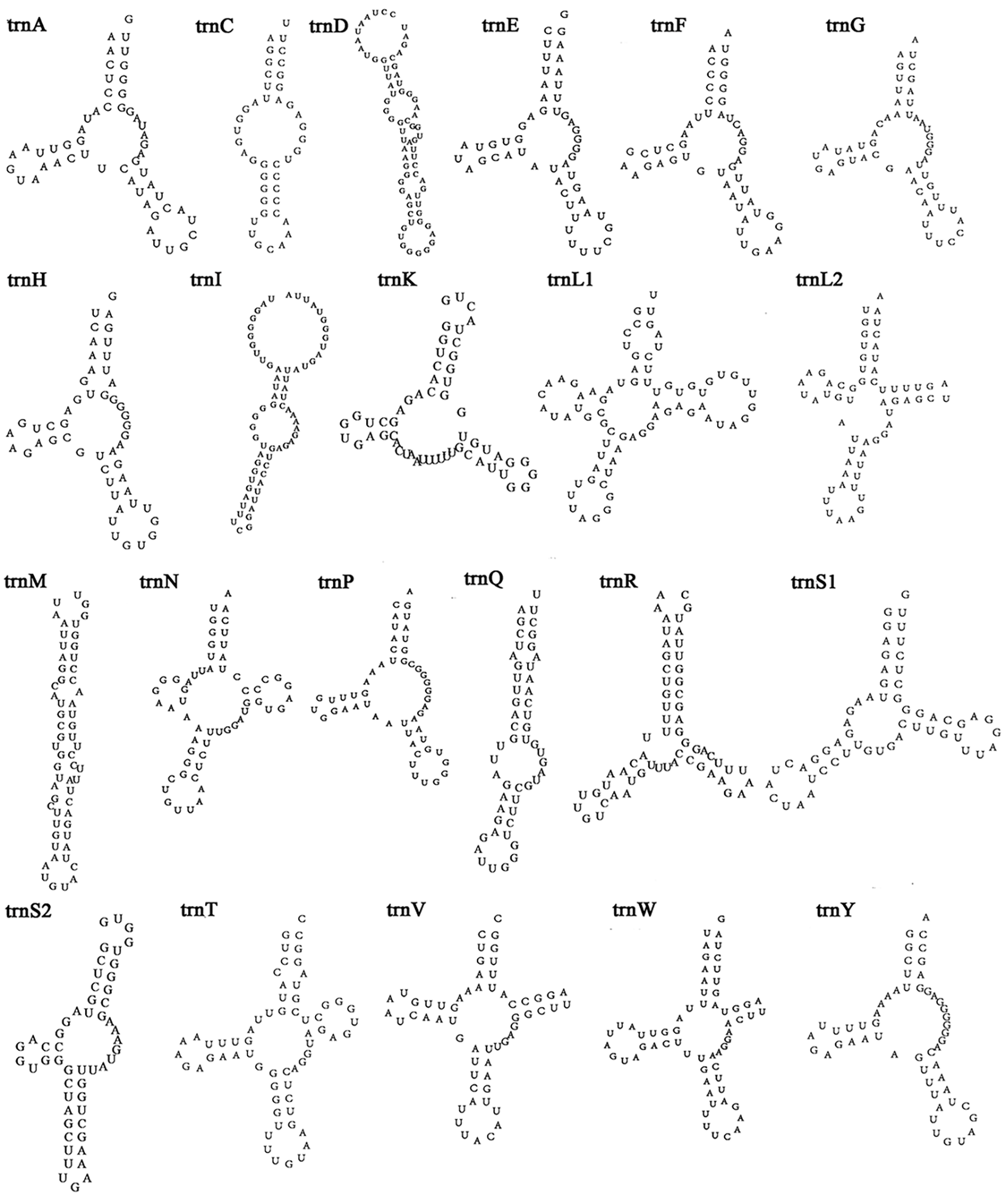

Supplement: Ren et al. supplementary material 1 — Ren et al. supplementary material [file S003118202510036Xsup001.tiff]

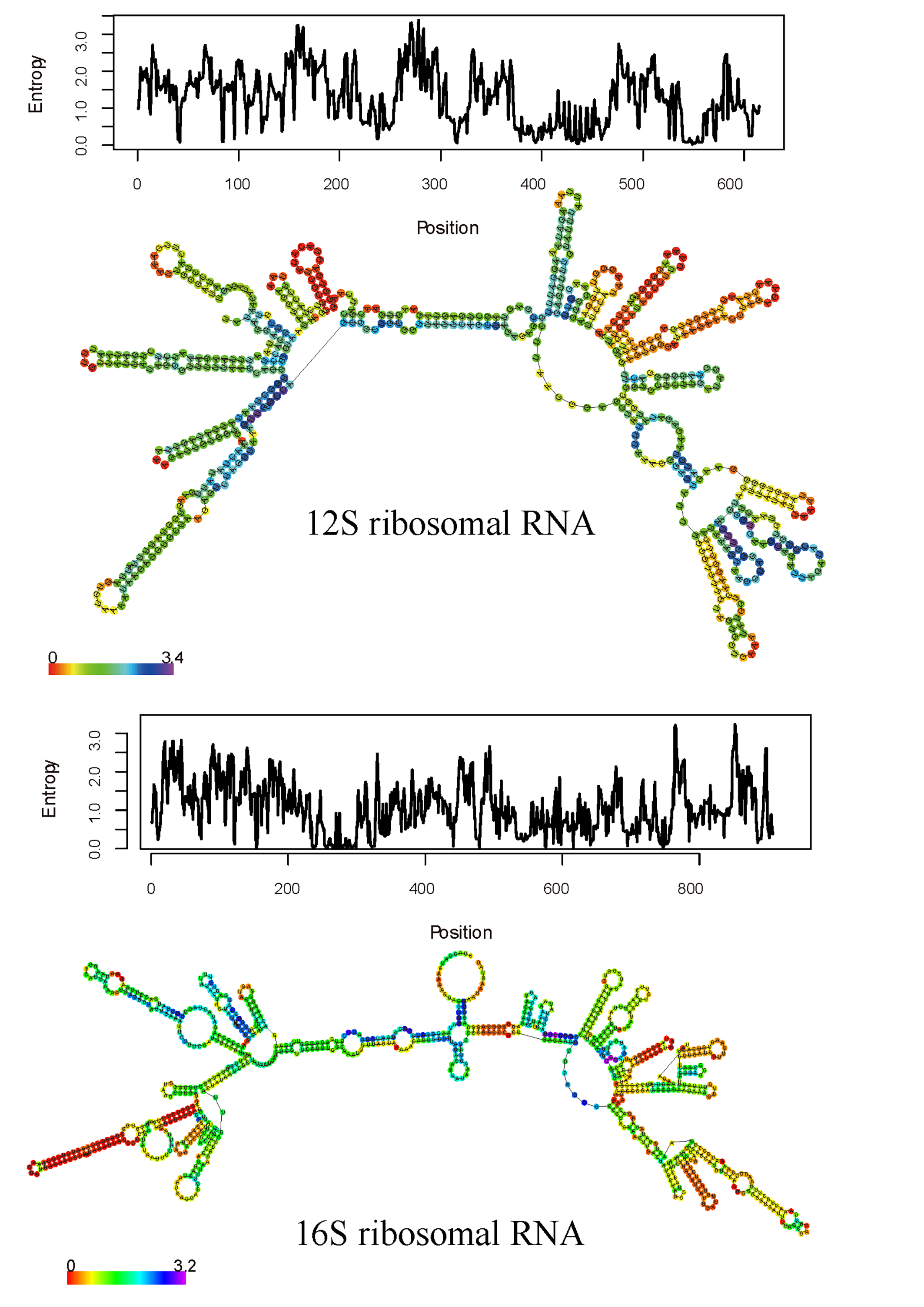

Supplement: Ren et al. supplementary material 2 — Ren et al. supplementary material [file S003118202510036Xsup002.tiff]

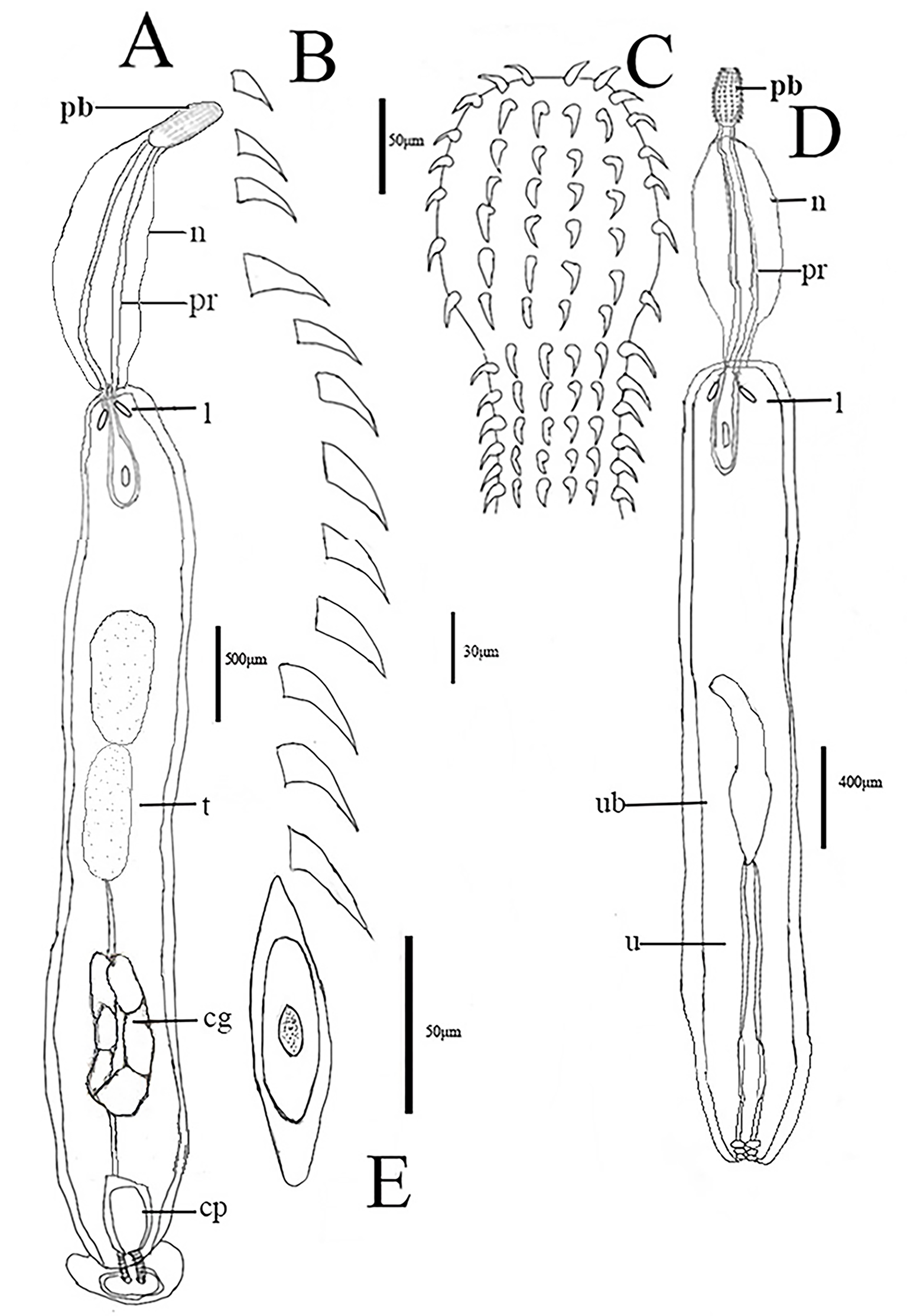

Supplement: Ren et al. supplementary material 3 — Ren et al. supplementary material [file S003118202510036Xsup003.tiff]
