# Supplementary material for: Characterization of the complete mitochondrial genome of Longicollum pagrosomi yamaguti, 1935 (Palaeacanthocephala: Echinorhynchida) in cultured large yellow croaker (Larimichthys crocea) and its phylogenetic implications
Source: Parasitology. 2025 Jul 1;152(9):951–7. doi: 10.1017/S003118202510036X (PMC12644934; doi:10.1017/S003118202510036X)
Supplement: Ren et al. supplementary material 4 — Ren et al. supplementary material [file S003118202510036Xsup004.docx]

**Table S1.** Comparative morphometric data for *Longicollum pagrosomi*.

| **Country** | Ningde, China | | Pingtung, China | | China (East China Sea) | | Japan (Inland Sea) |
| --- | --- | --- | --- | --- | --- | --- | --- |
| **Source** | *L. crocea* | | *L. erythropterus* | | *O. fasciatus* | | *P. auratus* |
| **Characteristic** | Male | Female | Male | Female | Male | Female | Not stated |
| **Trunk length (mm)** | 12.96~17.16 | 10.96-17.82 | 10.12-14.33 | 9.18-14.86 | 9.00-12.4 | 7.90-11.4 | 12.0-17.0 |
| **Trunk width (mm)** | 1.34-1.88 | 1.46-2.77 | 0.89-1.33 | 0.83-1.52 | 0.95-1.29 | 0.98-1.31 | Not stated |
| **Neck length (mm)** | 2.43-4.39 | 2.20-5.57 | 4.08-5.76 | 4.11-5.91 | 3.78-4.24 | 3.53-5.13 | up to 5.00 |
| **Neck width (mm)** | 0.85-1.26 | 0.86-1.87 | Not stated | Not stated | 0.66-0.80 | 0.57-0.68 | up to 1.50 |
| **Proboscis length (mm)** | 0.77-1.38 | 1.08-1.13 | 0.87-1.05 | 0.80-1.09 | 0.50-0.73 | 0.63-0.83 | 0.90-1.30 |
| **Proboscis width (mm)** | 0.40-0.49 | 0.40-0.54 | 0.40-0.56 | 0.38-0.48 | 0.35-0.46 | 0.39-­0.53 | 0.50-0.60 |
| **HLR** | 10-14 | 10-14 | 9-12 | 9-12 | 9-12 | 9-12 | 9-12 |
| **LH** | 11-16 | 11-16 | 11-14 | 11-14 | 14 | 14 | 11-15 |
| **Hook length (μm)** | 22-39 | 21-43 | 31-40 | 29-39 | 34-48 | 29-43 | 45-60 |
| **PRL (mm)** | 4.85-7.33 | 5.87-6.45 | 4.57-6.11 | 4.75-6.48 | 5.05-6.38 | 4.70-6.73 | Not stated |
| **Testis length (mm)** | 0.88-1.48 |  | 0.83-1.41 |  | 0.78-1.29 |  | up to 1.50 |
| **Uterine bell (mm)** |  | 0.28-0.35 |  | 0.15-0.30 |  | 0.12-0.24 | Not stated |
| **NCG** | 6 | 6 | 6 | 6 | 6 | 6 | 6 |
| **Egg length (μm)** |  | 11-38 |  | 53-68 |  | 60-72 | Not stated |
| **Egg width (μm)** |  | 6-12 |  | 15-20 |  | 12-17 | Not stated |
| **Reference** | This study | | Cheng et al., 2021 | | Li et al., 2017 | | Yamaguti, 1935 |

Abbreviations: HLR, hooks per longitudinal row; LH, longitudinal rows of proboscis hooks; PRL, proboscis receptacle length; NCG, number of cement glands
