# Supplementary material for: Characterization of the complete mitochondrial genome of Longicollum pagrosomi yamaguti, 1935 (Palaeacanthocephala: Echinorhynchida) in cultured large yellow croaker (Larimichthys crocea) and its phylogenetic implications
Source: Parasitology. 2025 Jul 1;152(9):951–7. doi: 10.1017/S003118202510036X (PMC12644934; doi:10.1017/S003118202510036X)
Supplement: Ren et al. supplementary material 5 — Ren et al. supplementary material [file S003118202510036Xsup005.docx]

**Table S2**. Primers used for amplification of the *Longicollum pagrosomi* mitogenome.

| **Fragment No.** | **Gene or region** | **Primer name** | **Sequence (5’-3’)** | **Length (bp)** |
| --- | --- | --- | --- | --- |
| F1 | 16S | ETF1 | GTTGACTATGCTAAGGTAGC | 336 |
|  |  | ETR1 | CTTACACCGATCTAAACTCA |  |
| F2 | 16S-NAD4 | ETF2 | GCTATCTAGCTAATAGCTAG | 4252 |
|  |  | ETR2 | CCTACAACCGCCCACACTCC |  |
| F3 | NAD4 | ETF3 | CTACCTAAAGTTCATGTGG | 223 |
|  |  | ETR3 | CCACTGAAGAGTAAGCAAC |  |
| F4 | NAD4-NAD5 | ETF4 | TGGTGTTAGCTGGGGTCGTC | 1170 |
|  |  | ETR4 | TCATGGCCACATCACCTACC |  |
| F5 | NAD5 | ETF5 | CTTTTAGGGTGGGAGTTCTTAGG | 294 |
|  |  | ETR5 | CACAGGTGTGGGGGCAGCCA |  |
| F6 | NAD5-CYTB | ETF6 | GTGGTGATGGTTGGATTGGT | 1822 |
|  |  | ETR6 | CTGTGAGCCCCAGCCCTAGG |  |
| F7 | CYTB | ETF7 | ATGGGCTATGTGCTACCTTG | 434 |
|  |  | ETR7 | TACCACTCAGGTTTGATATG |  |
| F8 | CYTB-12S | ETF8 | GGATTTTGTATGTGGTGTGGC | 1967 |
|  |  | ETR8 | CACAGGTATCTAATCTGCTTC |  |
| F9 | 12S | ETF9 | GGCTGCGGTTATTCAGCTAG | 401 |
|  |  | ETR9 | GGTATTGACGGGCGATATGTAC |  |
| F10 | 12S-16S | ETF10 | CAAATTCCATAAGGGTTGGAAG | 5079 |
|  |  | ETR10 | GCAATTTCACTTCGCTACA |  |
