# Supplementary material for: Characterization of the complete mitochondrial genome of Longicollum pagrosomi yamaguti, 1935 (Palaeacanthocephala: Echinorhynchida) in cultured large yellow croaker (Larimichthys crocea) and its phylogenetic implications
Source: Parasitology. 2025 Jul 1;152(9):951–7. doi: 10.1017/S003118202510036X (PMC12644934; doi:10.1017/S003118202510036X)
Supplement: Ren et al. supplementary material 6 — Ren et al. supplementary material [file S003118202510036Xsup006.docx]

**Table S3.** Detailed information of species used for phylogenetic analyses.

| **Order** | **Family** | **Species** | **Size (bp)** | **Accession no.** |
| --- | --- | --- | --- | --- |
| Echinorhynchida | Pomphorhynchidae | *L**ongicollum pagrosomi* | 14632 | OR215045 |
|  |  | *Pomphorhynchus sp. TP-2012* | 13878 | JQ824372 |
|  |  | *Pomphorhynchus laevis* | 13772 | NC060708 |
|  |  | *Pomphorhynchus rocci* | 13845 | NC060484 |
|  |  | *Pomphorhynchus bulbocolli* | 13915 | NC060483 |
|  |  | *Pomphorhynchus tereticollis* | 13891 | NC060482 |
|  | Pseudoacanthocephalidae | *Pseudoacanthocephalus sp* | 14883 | OQ588705 |
|  | Leptorhynchoididae | *Brentisentis yangtzensis* | 13864 | MK651258 |
|  | Rhadinorhynchidae | *Leptorhynchoides thecatus* | 13888 | NC006892 |
|  | Echinorhynchidae | *Echinorhynchus truttae* | 13659 | NC019805 |
| Philodinida | Philodinidae | *Philodina citrina* | 14003 | FR856884 |
| Bdelloida | Bdelloidea | *Rotaria rotatoria* | 15319 | NC013568 |
| Oligacanthorhynchida | Oligacanthorhynchidae | *Macracanthorhynchus hirudinaceus* | 14282 | NC019808 |
|  |  | *Oncicola luehei* | 14281 | NC016754 |
| Gyracanthocephala | Quadrigyridae | *Acanthogyrus cheni* | 13695 | KX108947 |
|  |  | *Pallisentis celatus* | 13855 | NC022921 |
| Polyacanthorhynchida | Polyacanthorhynchidae | *Polyacanthorhynchus caballeroi* | 13956 | NC029766 |
| Neoechinorhynchida | Neoechinorhynchidae | *Hebesoma violentum* | 13393 | KC415004 |
|  | Paratenuisentis | *Paratenuisentis ambiguus* | 13574 | NC019807 |
| Polymorphida | Polymorphidae | *Southwellina hispida* | 14742 | NC026516 |
|  |  | *Centrorhynchus aluconis* | 15144 | KT592357 |
|  | Plagiorhynchidae | *Plagiorhynchus transversus* | 15477 | NC029767 |
